# Eosinophilic Angiocentric Fibrosis as a Stenosing Lesion in the Subglottis

**DOI:** 10.1155/2017/2381786

**Published:** 2017-01-30

**Authors:** Ivan Keogh, Rohana O'Connell, Sean Hynes, John Lang

**Affiliations:** ^1^Otorhinolaryngology Department, University College Hospital Galway and Academic Department of Otorhinolaryngology, National University of Ireland Galway, Newcastle Road, Galway, Ireland; ^2^Department of Pathology, University College Hospital Galway, Newcastle Road, Galway, Ireland

## Abstract

Subglottic Eosinophilic Angiocentric Fibrosis (EAF) is an extremely rare disease of an elusive aetiology. It is chronically progressive benign condition that causes narrowing of the subglottic region leading to dysphonia and airway compromise. The diagnosis is historical and imaging is nonspecific. We report a case xc of 56-year-old lady referred to our institution with globus sensation, hoarseness, and mild stridor. Incidental subglottic mass was found at time of diagnostic microlaryngoscopy and biopsy confirmed subglottic EAF. All laboratory investigations were unremarkable. Lesion was removed with laryngeal microdebrider and three courses of intravenous dexamethasone were administered. Patient's postoperative period was uneventful and had remained disease free for 1 year. To date, no consensus has been reached on the optimal treatment of subglottic EAF. We recommend regular follow-up to detect early recurrence.

## 1. Introduction 

Eosinophilic Angiocentric Fibrosis (EAF) had been described as rare submucosal fibrosing vasculitis, believed by some to be the mucosal counterpart of granuloma faciale [1]. Numerous cases have been reported to date, mainly involving the upper airway, and are particularly common at the nasal septum [2, 3].

To date, 51 cases have been reported in the English literature and majority of reported occurrences of this benign condition are in the nasal cavity [4]. Only three cases have been identified in the subglottic region.

Holmes and Panje were the first to describe this condition in 1983 followed by Roberts and McCann two years later, who reported two cases of female patients with rare stenosing lesion involving the upper airway and coined the descriptive diagnosis of* Eosinophilic Angiocentric Fibrosis* [1, 5]. Roberts and McCann also believed that it might be allergies associated with an environmental agent [1]. Recently, EAF has been classified as an IgG4-related disease (IgG4-RD) [2].

EAF typically presents in young to middle aged females, as slowly progressive upper airway obstruction. Although rare, involvement of other anatomic regions such as the orbits, larynx, and trachea has also been reported with patients presenting with diplopia, epiphora, and stridor [6–9].

We report a rare case of subglottic EAF and review of patient's clinical course, her radiological findings, historical diagnosis, and treatment.

## 2. Case Report

A 58-year-old lady was referred to us with 2 years' history of hoarseness and globus sensation in her throat. She had no dysphagia or odynophagia but reported slowly progressive noisy breathing. She had no previous history of any surgery requiring intubation and no allergies or atopy or aspirin sensitivity. Mild audible biphasic stridor was noted. Oropharyngeal examination was normal. Fibreoptic nasoendoscopy revealed asymmetry of the glottis and limited view of the subglottis. Chest X-ray was normal.

On the basis of these findings and to further evaluate her airway, microlaryngoscopy was arranged. Difficulties were encountered during anaesthesia with poor visualisation during intubation leading to trauma to an incidental lesion in the subglottic region. After successful intubation with microlaryngeal tube, the subglottic mass was thoroughly assessed by the senior author. Biopsies were taken and haemostasis was ensured. Intraoperative intravenous dexamethasone was administered and patient was extubated safely. The rest of her postoperative course was uneventful.

**Figure 1:**
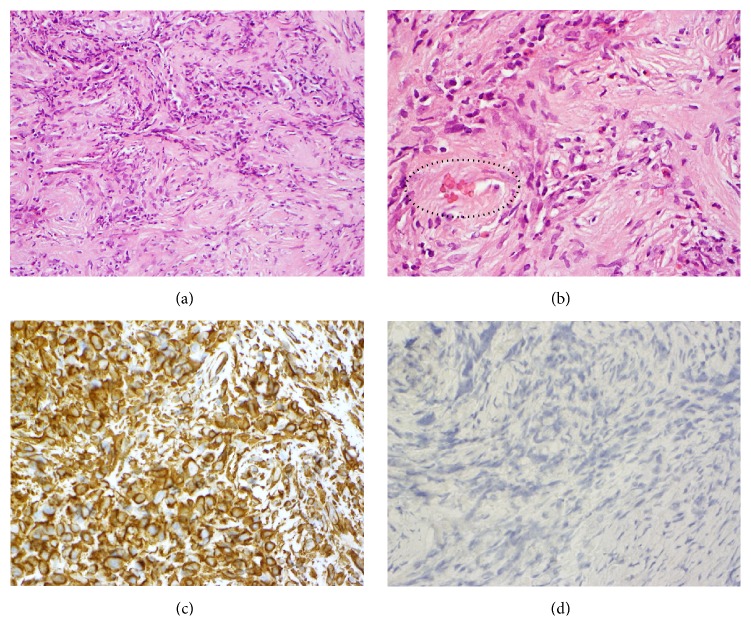
(a) Histology of the dense collagenous sclerosing lesion with acute and chronic inflammatory cells (10x objective). (b) Histology demonstrating significant numbers of eosinophils in the inflammatory infiltrate which surrounds a vessel (circled) (40x objective). (c) Positive staining of the lesion's cells for vimentin with immunohistochemistry (40x objective). (d) Negative staining for S100 and AE1/AE3 (40x objective).

Histology of the lesion under low power view (20x objective) revealed dense collagenous sclerosing lesion with acute and chronic inflammatory cells and examination under high power (40x objective) revealed significant numbers of eosinophil in the inflammatory infiltrate which surrounds vessels (see Figure 1). Lesion also showed positive staining for vimentin and negative for S100 and AE1/AE3 (see Figure 1).

**Figure 2:**
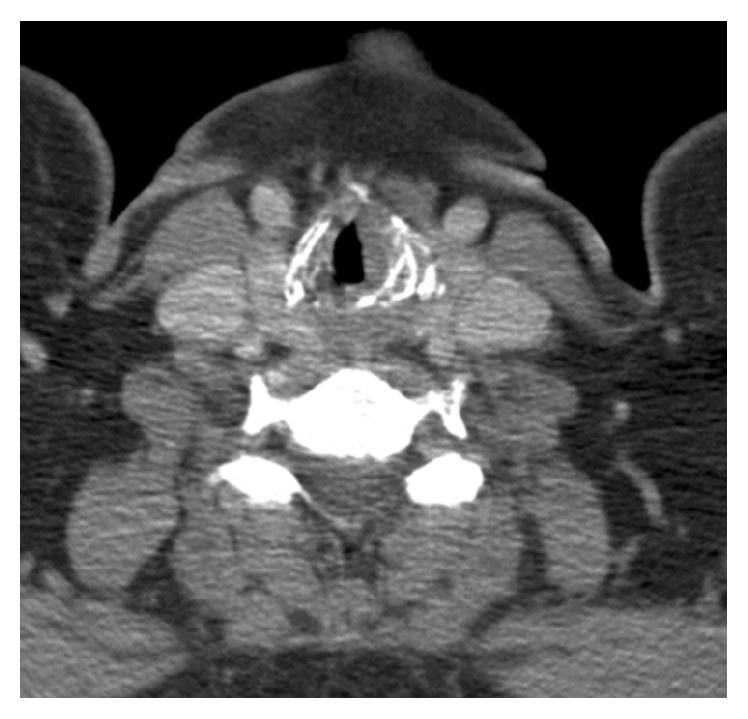
Axial section of CT scan showing left-sided laryngeal asymmetry and homogenous soft tissue mass in the subglottic region.

**Figure 3:**
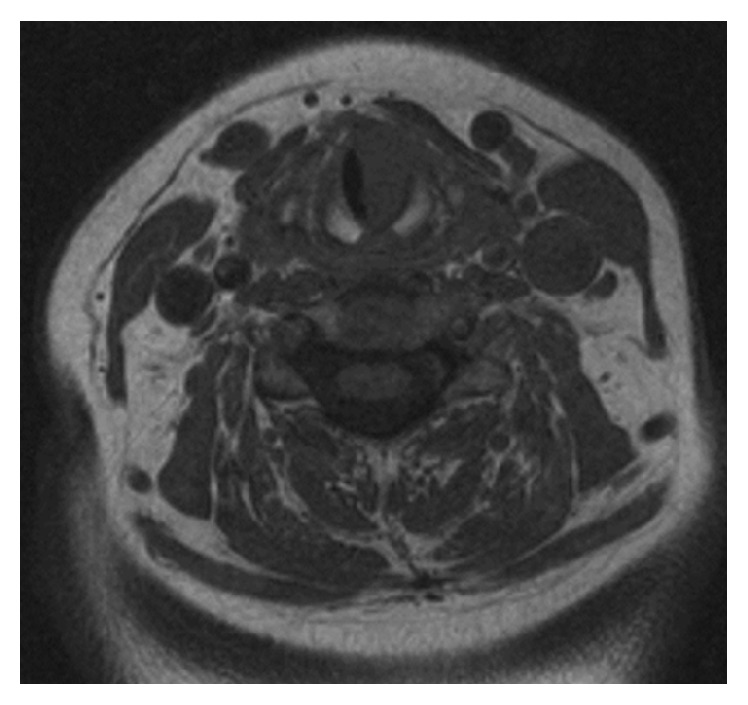
T1-weighted MRI scan showing homogenous soft tissue mass in the left subglottic region.

Imaging with CT scan that was carried out two weeks after biopsy confirmed laryngeal asymmetry; the lesion appeared homogenous with no surrounding cartilaginous or bony invasion (see Figure 2). MRI confirmed soft tissue mass approximately 1 cm in diameter confined to the subglottic region (see Figure 3).

Laboratory investigations, including routine blood examinations, blood biochemistry, and erythrocyte sedimentation rate (ESR), and coagulation parameters were all within the normal ranges. Antinuclear antibody (ANA), anti-PR3 antibody (c-ANCA), and anti-MPO antibody (p-ANCA) were also within the normal range.

**Figure 4:**
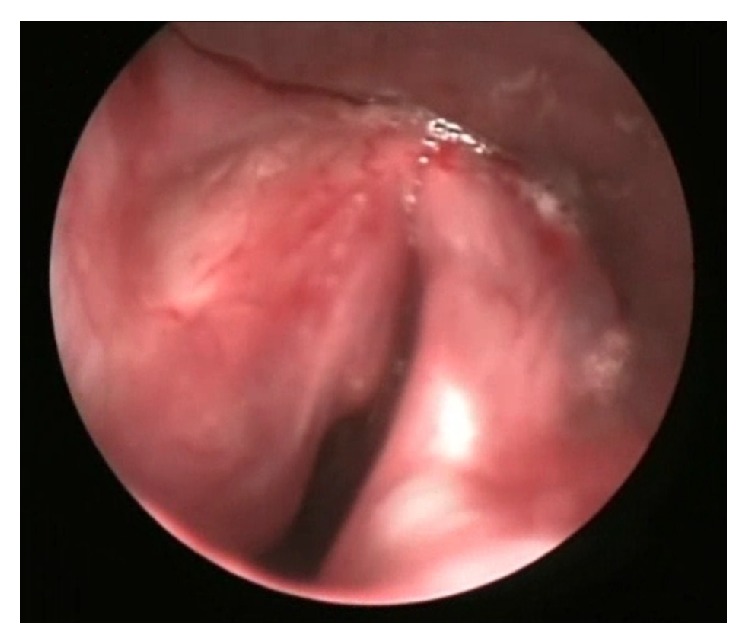
View of the glottis and superior aspect of the subglottic mass.

This case was discussed in the multidisciplinary meeting. Diagnosis of EAF was confirmed and the need for definitive management was acknowledged. Patient was booked in for definitive surgery 6 weeks after biopsy. This time precautions were taken not to traumatise the lesion and vocal cords during intubation with microlaryngeal tube (see Figure 4). Initially submucosal flap was elevated and laryngeal microdebrider was used to remove the tumour. Minimal bleeding was encountered and all macroscopic tumours were removed successfully.

Patient received dexamethasone intraoperatively and two further doses 8 hours apart. Her postoperative period was uneventful and was discharged home the following day. She was seen subsequently in the outpatient clinic, with improvement in her breathing and resolution of her stridor.

We recommend long term clinical follow-up, based on patient's symptoms and clinical examination with fibreoptic nasoendoscopy. So far, one year after surgery, there has been no sign or symptoms of local recurrence.

## 3. Discussion

Subglottic EAF is an exceedingly rare condition with no definitive aetiology. Three cases of subglottic EAF had been reported previously. The first case was reported by Roberts and McCann, of a 33-year-old female with asymptomatic subglottic stenosis noted at intubation [1]. This patient was successfully treated with cricotracheal resection [1]. Fageeh et al. reported the case of a 25-year-old female with history of progressive dyspnea [9]. Imaging revealed subglottic narrowing [9]. This patient had negative serological (CBC, ESR, and ANCA) studies [9]. She was initially treated with tracheotomy and dilatation and was given Tamoxifen [9]. Ultimately, she underwent cricotracheal resection for definite management [9]. The third case of subglottic EAF was reported by Nogueira et al. in 2011 [10]. The patient in this case report was a 68-year-old female who presented with nasal plaques, hoarseness, and dyspnea. Serological test revealed eosinophilia (6.7%) and mildly elevated ESR. CT scan revealed a concentric subglottic narrowing. This patient underwent wide local excision of the subglottic lesion.

EAF shares similar histological features to granuloma faciale of the skin and sometimes can occur concurrently in association with this benign skin condition, as reported by Nogueira et al. [10]. The histology of EAF is pathognomonic and is characterised by progression from an early eosinophil-rich perivascular fibrosing inflammatory lesion to a late dense perivascular “onion-skin” fibrosis formation with decreased inflammatory infiltration [11, 12]. Eosinophils are the predominant inflammatory cells [11, 12]. The main histological differential diagnosis includes granuloma faciale, Wegener's granulomatosis, Churg-Strauss syndrome, and Kimura's disease [13]. All these lesions have prominent eosinophil infiltrates. Negative blood test for c-ANCA and p-ANCA excludes Wegener's granulomatosis and Churg-Strauss syndrome while absence of dense lymphoid aggregates with prominent germinal centres exclude Kimura's disease [13].

Based on most literature, the average duration of clinical symptoms ranged from 3 to 6 years, with majority experiencing symptoms for more than 4 years [1, 9, 14]. Our patient suffered for almost 2 years with globus sensation, hoarseness, and stridor. The long history of the symptoms suggests chronically progressive upper airway obstructive disease resulting in substantial narrowing of the subglottic region. Therefore, it is crucial for subglottic EAF to be evaluated and confirmed historically.

The radiological findings of subglottic EAF in our case were nonspecific and included soft tissue mass in the subglottic region. There was no evidence of focal bony or cartilaginous erosion. On nonenhanced CT scan, the subglottic lesion appeared homogenous and similar finding was also noted on T1-weighted MRI scan. The characteristic whorled “onion-skin” collagenous tissue that is usually observed in the late stage on T2-weighted MRI scan was not seen.

To date, no consensus has been reached on the treatment strategy of EAF, let alone on subglottic EAF. It has been generally accepted that EAF lacks malignant degeneration with no potential for distant metastasis. But it still has the ability to cause focal destruction leading to organ malfunction. Slowly progressive EAF in the subglottic region has the potential to cause dysphonia, dysphagia, and life threatening airway obstruction. The most common treatment modality of EAF in most case reports is surgical resection [4, 6, 7, 11, 14]. The recurrence rate reported is extremely high with approximately 70% of patients experiencing persistence disease following treatment [4].

In most instances, medical treatment has not been effective, although few reported symptomatic relief. Fageeh et al. reported good results with intralesional injection of subglottic EAF [9]. Surgical resections of EAF located elsewhere have resulted in disease-free follow-up in approximately 30% of patients [15]. Majority of patients with recurrences still require multiple resections. Nogueira et al. combined both surgical and medical therapies in treatment of concurrent granuloma faciale on the face and subglottic EAF [10]. He reported excellent response to an intralesional corticosteroid on the GF lesion, CO2 laser on the EAF lesion, and oral dapsone treatment [10].

Regular and longer follow-ups are necessary to confirm surgical completeness. Our patient has remained disease free for almost 1 year now with no evidence of disease recurrence and is completely asymptomatic.

In conclusion, subglottic EAF is an exceedingly rare and chronically progressive disease with an elusive aetiology. Though benign, subglottic EAF has the potential to cause dysphonia, dysphagia, and airway obstruction. It has historical diagnosis and radiological findings are often nonspecific. Treatment remains a challenge, necessitating regular follow-up after surgery to confirm surgical completeness.
